# HBI‐8000 improves heart failure with preserved ejection fraction via the TGF‐β1/MAPK signalling pathway

**DOI:** 10.1111/jcmm.18238

**Published:** 2024-03-20

**Authors:** Jing Tian, Wenjing Li, Lu Zeng, Yang Li, Jiamin Du, Ying Li, Bin Li, Guohai Su

**Affiliations:** ^1^ Central Hospital Affiliated to Shandong First Medical University Jinan Shandong China; ^2^ Research Center of Translational Medicine, Jinan Central Hospital Shandong First Medical University Jinan Shandong China; ^3^ Department of Cardiology, Jinan Central Hospital, Cheeloo College of Medicine Shandong University Jinan Shandong China

**Keywords:** angiotensin II, cardiac fibrosis, HBI‐8000, heart failure with preserved ejection fraction, TGF‐β1/MAPK signalling pathway

## Abstract

Heart failure with preserved ejection fraction (HFpEF) accounts for approximately 50% of total heart failure patients and is characterized by peripheral circulation, cardiac remodelling and comorbidities (such as advanced age, obesity, hypertension and diabetes) with limited treatment options. Chidamide (HBI‐8000) is a domestically produced benzamide‐based histone deacetylase isoform‐selective inhibitor used for the treatment of relapsed refractory peripheral T‐cell lymphomas. Based on our in vivo studies, we propose that HBI‐8000 exerts its therapeutic effects by inhibiting myocardial fibrosis and myocardial hypertrophy in HFpEF patients. At the cellular level, we found that HBI‐8000 inhibits AngII‐induced proliferation and activation of CFs and downregulates the expression of fibrosis‐related factors. In addition, we observed that the HFpEF group and AngII stimulation significantly increased the expression of TGF‐β1 as well as phosphorylated p38MAPK, JNK and ERK, whereas the expression of the above factors was significantly reduced after HBI‐8000 treatment. Activation of the TGF‐β1/MAPK pathway promotes the development of fibrotic remodelling, and pretreatment with SB203580 (p38MAPK inhibitor) reverses this pathological change. In conclusion, our data suggest that HBI‐8000 inhibits fibrosis by modulating the TGF‐β1/MAPK pathway thereby improving HFpEF. Therefore, HBI‐8000 may become a new hope for the treatment of HFpEF patients.

## 1 INTRODUCTION

Heart failure with preserved ejection fraction (HFpEF) is a clinical syndrome characterized by ventricular diastolic dysfunction, the incidence of which continues to rise with the aging of the population and the increase in the number of people with diabetes, hypertension and obesity.^1^ A few studies on myocardial biopsies of patients with HFpEF have shown that myocardial hypertrophy, interstitial fibrosis, coronary microvascular rarefaction, systemic inflammation and oxidative stress may be involved in the pathophysiological process of HFpEF,^2^, ^3^, ^4^ in which myocardial hypertrophy and fibrosis cause diastolic insufficiency of the heart, which is an important cause of the development of HFpEF. Therefore, inhibiting cardiac hypertrophy and fibrosis can have a significant impact on HFpEF.

Cardiac fibrosis is a pathological manifestation characterized by proliferation of cardiac fibroblasts (CFs), phenotypic transformation of myofibroblasts, increased collagen secretion and excessive deposition of extracellular matrix (ECM), and it is a common pathological change in the advanced stages of many cardiovascular diseases.^5^ In normal circumstances, CFs are quiescent and are primarily responsible for maintaining collagen synthesis and catabolism in the ECM.^6^ As a result of various pathological factors, CFs are activated and differentiate into myofibroblasts (MFs) with enhanced ability to proliferate, migrate, contract, and produce ɑ‐smooth muscle actin (ɑ‐SMA).^7^, ^8^ MFs secrete large amounts of ECM (e.g. Col1 and Col3),^9^ and ECM is overdeposited in the interstitium and perivascular areas of the heart, which can lead to myocardial ventricular wall stiffness, reduced compliance and enlarged cardiac chambers, causing cardiac dysfunction. Therefore, inhibition of the conversion of CFs to MFs is a key component of antifibrotic therapy. During the development of cardiac fibrosis, transforming growth factor‐β1 (TGF‐β1) is the main pro‐fibrotic factor that mediates the conversion of CFs to MFs. There is evidence that TGF‐β1/MAPK pathway activation is associated with myocardial fibrosis and heart failure.^10^ Mitogen‐activated protein kinase (MAPK) are comprised of three subfamilies, namely extracellular signal‐regulated kinases (ERKs), c‐Jun N‐terminal kinases (JNKs) and p38MAPKs,^11^ which can be activated by pathological factors to recruit RASS to promote CFs proliferation and transcription of pro‐fibrotic factors.^12^

In recent years, the regulation of histone acetylation and deacetylation in the field of epigenetics has received extensive attention in the cardiovascular field. Histone acetylation is mainly regulated by histone acetyltransferase, which promotes the transcription of relevant genes by dissociating histone‐bound genomic DNA, while histone deacetylase (HDAC) acts in the opposite way, which inhibits the transcription of target genes by removing acetylated residues on lysines and re‐densifying the chromatin structure. Histone deacetylases inhibitors (HDACi) are a class of organic compounds that inhibit histone deacetylation, and they have been used in a large number of applications in the treatment of oncological diseases. Clinical studies have shown that HDACi plays an important role in the pathophysiology of a variety of cardiovascular diseases, including myocardial hypertrophy, hypertension, atherosclerosis, and heart failure.^12^, ^13^, ^14^, ^15^, ^16^, ^17^ Chidamide (HBI‐8000) is a subtype‐selective oral HDACi that can selectively inhibit HDAC1, 2, 3 and 10. It has a good safety profile, as well as significant efficacy against a variety of malignant tumours.^18^ However, HBI‐8000 is insufficiently studied in cardiovascular disease and its effects on cardiac hypertrophy and fibrosis are unclear.

This study aimed to determine the therapeutic effects of HBI‐8000 in HFpEF and its related mechanisms. It was found that the model group of mice displayed hypertrophy and fibrotic changes, and HBI‐8000 administration significantly improved these pathological changes; at the cellular level, we found that HBI‐8000 was capable of inhibiting AngII‐induced cardiac fibrosis through the TGF‐β1/MAPK pathway. In conclusion, our data suggest that HBI‐8000 can improve HFpEF by inhibiting fibrosis through modulation of the TGF‐β1/MAPK pathway.

## 2 MATERIALS AND METHODS

### 2.1 Materials

#### 2.1.1 Experimental animal

Forty 6–7 weeks old SPF grade C57BL/6 male mice, body mass (20 ± 2) g, were purchased from Beijing Viton Lihua Laboratory Animal Co., Ltd. The temperature of the animal rearing room was 23°C, the relative humidity was 40% and the animals were reared under alternating darkness and light (12 h of light/12 h of darkness), with free access to food and water. The experiments involving animals were conducted in accordance with the relevant regulations of the Experimental Animal Ethics Committee at the Kechuang Centre of Shandong First Medical University.

#### 2.1.2 Drugs and reagents

HBI‐8000 (HY‐109015) was purchased from MedChemExpress (USA). Trizol (AG21102), Evo M‐MLV Reverse Transcription Premixed Kit (AG11728) and SYBR Green Pro Taq HS Premixed qPCR Kit (AG11728) were obtained from Aikorui Bioengineering Co., Ltd (Hunan, China). Masson Trichrome Staining Kit (G1340), HE Staining Kit (G1120), Modified Sirius Red Staining Kit (G11472) and 4% Cell Tissue Fixative were purchased from Beijing Solarbio Science & Technology Co., Ltd.

Antibodies against phospho‐p38MAPK (4511S), phospho‐JNK (4668S), phospho‐ERK (4370S) and ERK (4695S) were obtained from Cell Signaling Technology (Danvers, MA, USA). Antibodies against a‐SMA (A1011) were obtained from Abcam (Shanghai, China). Antibodies against TGF‐β1 (21989‐1‐AP), JNK (51151‐1‐AP), p38MAPK (14064‐1‐AP), proliferating cell nuclear antigen (PCNA, 60097‐1‐lg) and β‐actin (66009‐1‐lg) were obtained from Proteintech (Wuhan, China). SB203580 (p38MAPK inhibitor) was purchased from Sigma‐Aldrich (Shanghai, China). A cell counting kit‐8 assay (GK3607‐500T, DINGGUOCHANGSHENG Biotechnology, Beijing, China), Cell‐Light EdU Apollo567 In Vitro Kit (100T, C10310‐1, Ruibo, Guangzhou, China) and Actin‐Tracker Green (C1033, Beyotime Biotechnology, Shanghai, China) were used in this study.

### 2.2 Methods

#### 2.2.1 Animal grouping and modelling

A total of 40 C57BL/6N male mice aged 6–7 weeks were acclimatized and fed for 1 week before being randomly divided into two groups based on their body mass: a control group (Chow) and a model group (HFpEF), 10 and 20 mice in each group, respectively. The control group was given a regular diet, whereas the model group was given a mixed diet consisting of 60 kcal% high‐fat diet (HFD, D12492, China) and N‐Nitro‐L‐arginine methyl ester hydrochloride (L‐NAME, Sigma, N5751) (0.5 g/L, added to water).^19^ We performed a cardiac ultrasound 5 weeks after the HFpEF diet was started, mainly to determine left ventricular diastolic function (E/A).

Following the successful establishment of the mouse HFpEF model previously described, each group was randomized to receive either HBI‐8000 (0.1 mg/kg, 3 times/week for 4 weeks) or saline gavage.

#### 2.2.2 Ultrasound detection of mouse heart

FUJIFILM Vevo 3100LT system and MX250 probe were used to detect ultrasound. Pre‐detection chest hair removal was performed to expose the overall chest skin. The mice were anesthetized (maintenance oxygen flow 2.0/min, isoflurane 1.5%–2.0%), maintained at 38.0°C, and their heart rate was 400–500 beats per minute. The left ventricular long‐axis activity images were obtained in Parasternal long‐axis views in B‐Mode, and the mitral flow spectra were obtained in pulse‐wave mode in the apical four chambers at the maximum opening of the mitral valve. An ultrasound analysis was performed using Vevo LAB 3.2.6, and an average of at least five cycles of systolic and diastolic function was recorded.

#### 2.2.3 Mouse caudal artery blood pressure monitoring

The blood pressure of experimental mice was monitored using a non‐invasive CODA tail‐artery blood pressure monitor (Softon, BP‐2010A). Observations of the blood pressure change images were made, and volume >20 and standard images were selected as valid images of the blood pressure. Blood pressure was measured for three consecutive days at a fixed time, and the stable average value on the third or fourth day was taken as the mouse's blood pressure.

#### 2.2.4 Haematoxylin and eosin staining (HE staining), Masson staining and picric sirius red staining (PSR staining) to observe the histopathological changes in heart tissue

The heart tissues of mice in each group were taken, fixed in 4% paraformaldehyde for 24 h, routinely dehydrated, dipped in wax, embedded in paraffin and sectioned, stained with HE, Masson and PSR staining and sealed with neutral gum, respectively, and finally observed and photographed under a light microscope. Cell surface area was measured using Image‐Pro Plus 6.0 software.

#### 2.2.5 RNA extraction, reverse transcription and real‐time fluorescent quantitative PCR

Tissue RNA extraction: The cardiac tissue blocks were collected and homogenized in a 1.5 mL EP tube with 1 mL of Trizol and a certain number of beats until all the tissue blocks had been dissolved.

Cellular RNA extraction: (a) Discard the cell culture medium, wash with cooled PBS three times, and aspirate the supernatant. (b) According to the cell density, add Trizol, such as six‐well plate about 1 mL per well, scrape the cells with a cell scraper on ice, aspirate the supernatant in 1.5 mL EP tube. (c) Apply chloroform method to extract RNA and detect the concentration for subsequent experiments.

Real‐time fluorescence quantitative PCR was performed using the qRT‐PCR kit after reverse transcription using the reverse transcription kit. Three replicate wells were set up for each experiment and the experiment was repeated three times. Primers were designed using the Primer‐BLAST tool from the National Center for Biotechnology Information (NCBI), and the FASTA sequences were obtained from the gene database of the NCBI, with β‐actin as the internal reference gene and the primer sequences are shown in Table 1.

**TABLE 1:** Genes sequences.

	Gene name	Primer sequence (forward, reward)
Mouse	BNP	GCCTCACAAAAGAACACCCA
		CGATCCGGTCTATCTTGTGC
	β‐MHC	GCTCAGCAATCTATTTGCCAAC
		AGCCTTTCTTTGCCTTGCCT
	TGF‐β1	AGCTGCGCTTGCAGAGATTA
		AGCCCTGTATTCCGTCTCCT
	α‐*SMA(Acta2)*	TTCGTGACTACTGCCGAGC
		GTCAGGCAGTTCGTAGCTCT
	Col1a1	CCCTGGTCCCTCTGGAAATG
		GGACCTTTGCCCCCTTCTTT
	Col3a1	TGACTGTCCCACGTAAGCAC
		GAGGGCCATAGCTGAACTGA
	Fn1	AGTTTGTGCATGGTGTCCGA
		CAGTTGTGCCTGGGTAGGTC
	β‐Actin	GGCTGTATTCCCCTCCATCG
		CCAGTTGGTAACAATGCCATGT
Rat	α‐*SMA(Acta2)*	GGAGATATGGCGTGACTCACAA
		CGCTCAGCAGTAGTCACGAA
	Fn1	ACTGCAGTGACCAACATTGACC
		CACCCTGTACCTGGAAACTTGC
	TGF‐β1	TGACATGAACCGACCCTTCC
		TGTGGAGCTGAAGCAGTAGT
	Col1a1	CACTGCAAGAACAGCGTAGC
		AAGTTCCGGTGTGACTCGTG
	Col3a1	CAGCCTTCTACACCTGCTCC
		GTCGCCATTTCTCCCAGGAA
	β‐Actin	CTCTGTGTGGATTGGTGGCT
		CGCAGCTCAGTAACAGTCCG

#### 2.2.6 Protein extraction

Tissue protein extraction: (a) Obtain fresh heart tissue blocks, weigh and add SDS‐PAGE lysate (RIPA lysate: protease inhibitor: phosphatase inhibitor = 100:1:1) and a certain number of beats in a 1.5 mL EP tube, and then homogenize with a homogenizer until all the tissue blocks are lysed. (b) 12000 *g*, 4°C, centrifuge for 15 min and transfer the supernatant to a new EP tube. (c) Detect the protein concentration by BCA kit. (d) According to the protein concentration, add 5× Loading Buffer, so that the final concentration of protein sampling volume is 20 μg protein/10 μl total volume, and cook in a metal bath at 100°C for 10 min, and then freeze at −80°C for subsequent experiments.

Cell protein extraction: (a) Discard the cell culture medium, wash with cold PBS three times, and aspirate the supernatant. (b) Add SDS‐PAGE lysate according to the cell density (e.g. about 100ul per well for six‐well plates), scrape the cells with a cell scraper, and aspirate the supernatant in a 1.5 mL EP tube. (c) Centrifugation, detection of protein concentration, and cooking of proteins were carried out as described previously.

#### 2.2.7 Preparation of CFs and Neonatal rat cardiomyocytes (NRCMs)

1–3 days old wistar rats were taken, ventricles were cut and digested with digestive enzymes (HBSS buffer containing 200 U type II collagenase + 0.4% horse serum + 1% double antibody). The cell suspension was incubated in an incubator (37°C, 95% O_2_ and 5% CO_2_) for 1.15 h. NRCMs in the suspension were aspirated and BrdU was added to inhibit the proliferation of fibroblasts, and then replaced with normal myocardial medium after 72 h. Adherent fibroblasts were replaced with normal fibroblast medium and cultured in an incubator, and then digested with 0.25% trypsin and passaged at a ratio of 1:4 for subsequent experiments after 1–2 days.

#### 2.2.8 Cell proliferation assay

For the experimental procedure, homogeneous inoculations of CFs cells were made on 96‐well plates (2000 cells/100 μL), followed by a pretreatment with 5 mM HBI‐8000 for 1 h, followed by a treatment with 100 and 500 nM AngII for 24 h. Each group consisted of six replicate wells. For 1–4 h, the medium was replaced with normal medium containing 10% CCK‐8 assay solution. Then the absorbance of the samples at 450 nm was measured by an enzyme marker.

Additionally, we used the Cell‐Light EdU Apollo567 in vitro kit to detect CFs proliferation. The manufacturer's instructions were followed for specific experimental steps.

#### 2.2.9 Immunofluorescence

Immunofluorescence assay for ɑ‐SMA and PCNA content, the specific experimental procedure was carried out according to the manufacturer's instructions. Cells were analysed by a fluorescence microscope, and the results of signals were quantified by the software of ImageJ.

#### 2.2.10 Reactive oxygen species (ROS) measurement

Following Ang II treatment, intracellular ROS production in CFs were detected using a fluorescence microscope by using dichlorofluorescein diacetate (DCFH‐DA) staining. First, the CFs were incubated with 5 mM DCFH‐DA for 30 min at 37°C in the dark. Then, they were washed three times with serum‐free medium. ImageJ software was used to quantify the fluorescence intensity of each picture.

#### 2.2.11 Statistical analysis

The data are expressed as mean ± SEM. Statistical analyses were performed using SPSS 26.0 statistical software and Graphpad Prism 9.0 software. The statistical analysis of differences between two groups were assessed with the unpaired *t*‐test, and the differences among more than three groups were assessed by one‐way analysis of variation (ANOVA) followed by a Bonferroni's tests for post hoc analysis and multiple comparison tests. Differences with a *p* < 0.05 were considered statistically significant.

## 3 RESULTS

### 3.1 Establishment of HFpEF mouse models

**FIGURE 1:**
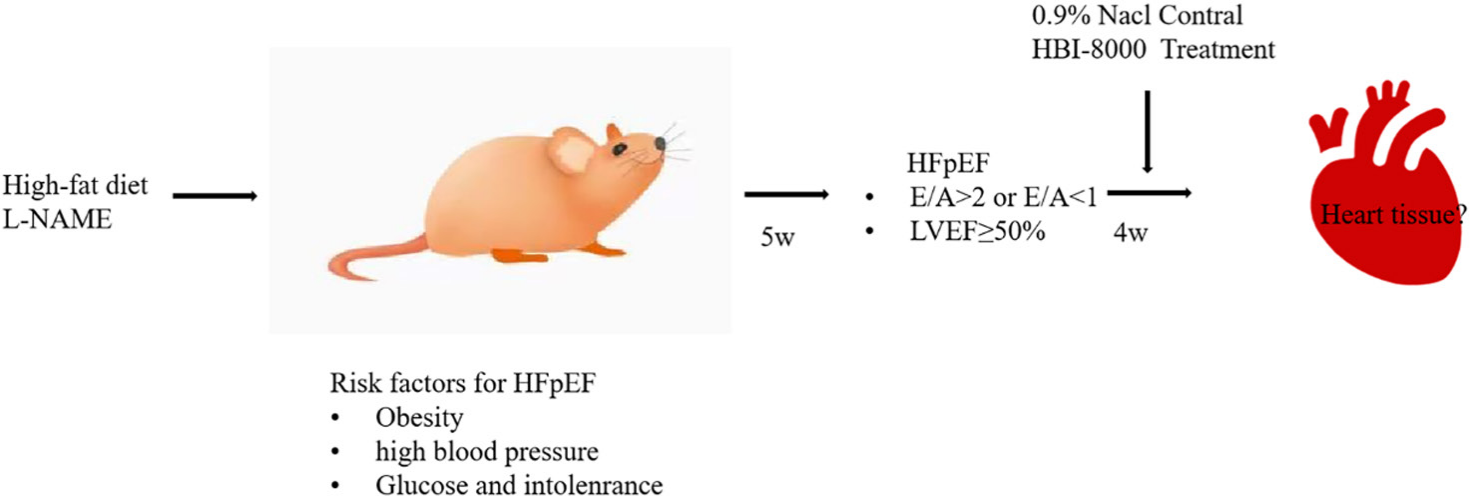
Flowchart for the experiment.

C57BL/6N male mice at 7–8 weeks of age were randomly divided into two groups; the Chow group was given a regular diet and the HFpEF group was fed a mixed diet for 5 weeks to induce the HFpEF phenotype. Below is a sketch of the experimental flow (Figure 1).

**FIGURE 2:**
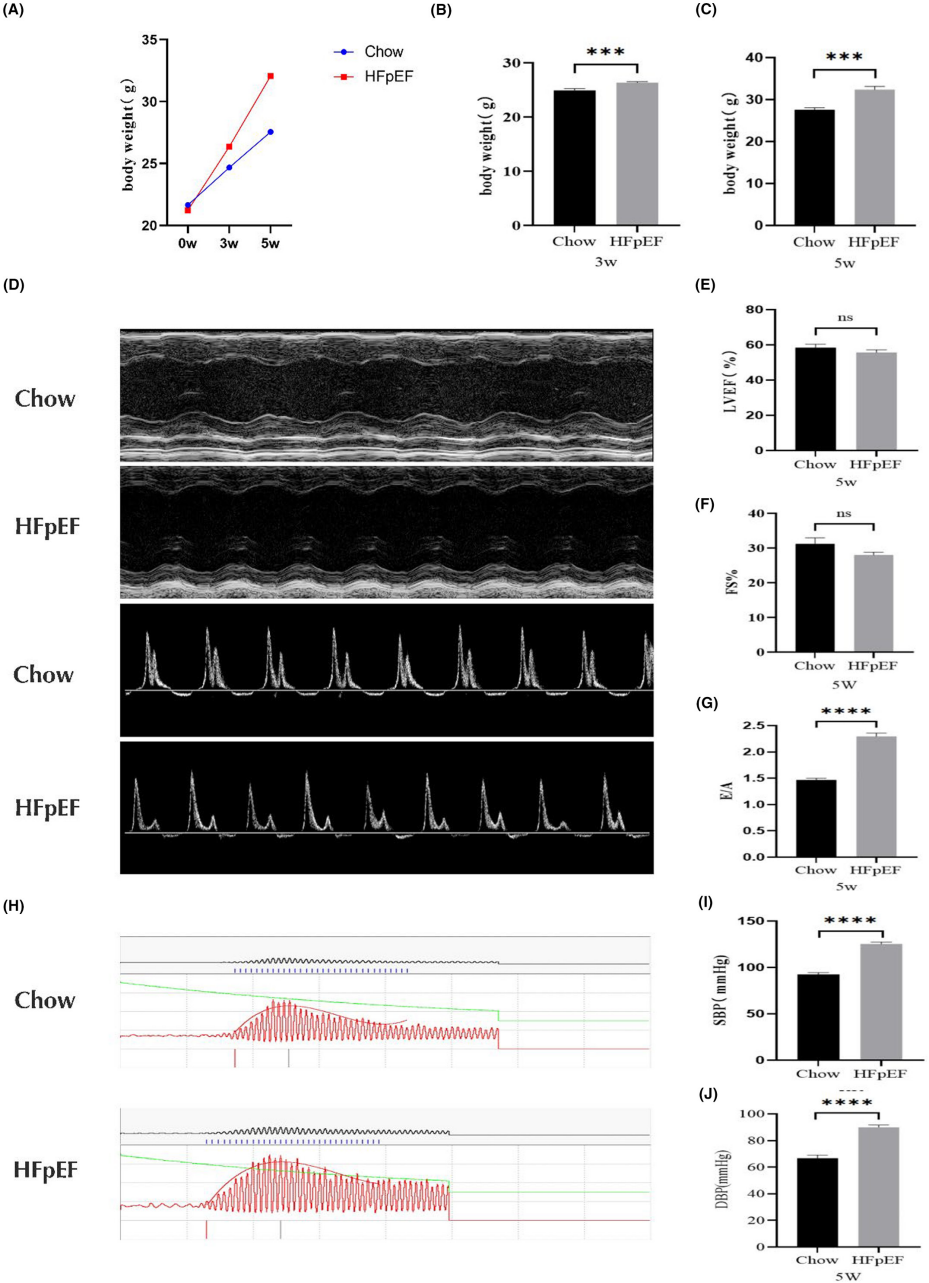
Various indices in mice during modelling. (A–C) Body weight levels of mice in each group during HFpEF modelling. Cardiac function of mice detected by cardiac ultrasound. (D) The upper half of the graph shows the characteristic M‐Mode image at Week 5, and the lower half shows the characteristic pulse‐wave image at Week 5. (E) Statistical data of LVFS in two groups of mice at Week 5. (F) is the LVEF statistics of two groups of mice at Week 5. (G) E/A statistics of two groups of mice at Week 5. (H–J) Blood pressure levels in the caudal artery of two groups of mice at Week 5 of modelling. *p < 0.05, **p < 0.01, ***p < 0.001, ****p < 0.0001. LVFS, left ventricular fractional shortening; LVEF, left ventricular ejection fraction; E, peak Doppler blood inflow velocity across mitral valve during early diastole; A, peak Doppler blood inflow velocity across mitral valve during late diastole. DBP, diastolic blood pressure; SBP, systolic blood pressure.

The results showed that the body weight of mice in the HFpEF group increased significantly with feeding time (Figure 2A–C). A statistical difference in body weight between the two groups of mice was observed by the third week of feeding (Chow 24.68 ± 0.98 g vs. HFpEF 27.56 ± 1.26 g). At Week 5 of modelling, the weight of mice in the HFpEF group had reached 32.38 ± 0.76 g compared to that of the Chow group (27.60 ± 0.49 g), and the results were statistically different.

The left ventricular fraction shortening (LVFS) and left ventricular ejection fraction (LVEF) were slightly lower in the HFpEF group of mice compared with the Chow group, but there was no statistically significant difference. (LVFS: Chow 31.23 ± 1.73 vs. 28.14 ± 0.86; LVEF: Chow 59.67 ± 2.48 vs. HFpEF 55.77 ± 1.45) (Figure 2D–F; Table 2). In addition, we examined left ventricular diastolic function in mice and showed that the E/A ratio was significantly elevated in HFpEF (Chow 1.47 ± 0.03 vs. HFpEF 2.22 ± 0.07) (Figure 2D,G; Table 2). The above data suggest that the HFpEF mixed diet has no significant effect on systolic function in mice, but severely impairs their diastolic function. In conclusion, the overall systolic‐diastolic function of the heart was decreased.

**TABLE 2:** Cardiac ultrasound data in mice.

	Chow (*n* = 10)	HFpEF (*n* = 20)
5 week diet		
HR (bpm)	504.13 ± 24.11	485.66.52 ± 19.19
LVID,d (mm)	3.40 ± 0.15	3.87 ± 0.07**
LVID,s (mm)	2.34 ± 0.13	2.83 ± 0.08**
LVAW,d (mm)	1.00 ± 0.09	1.07 ± 0.04
LVPW,d (mm)	0.92 ± 0.06	0.96 ± 0.04
LV Mass (mg)	139.88 ± 16.97	156.03 ± 5.56
LVFS (%)	31.23 ± 1.73	28.14 ± 0.86
LVEF (%)	59.67 ± 2.48	55.77 ± 1.45
Peak mitral E velocity (mm/s)	645.82 ± 37.99	724.91 ± 19.79
Peak mitral A velocity (mm/s)	362.16 ± 20.66	333.25 ± 12.72
Mitral E/A	1.47 ± 0.03	2.22 ± 0.07****

*Note*: Data are expressed as the means ± SEM. **p < 0.01, ****p < 0.0001.

Abbreviations: A, peak Doppler blood inflow velocity across mitral valve during late diastole; E, peak Doppler blood inflow velocity across mitral valve during early diastole; HR, heart rate; LVID,d, left ventricular internal diastolic diameter; LVID,s, left ventricular internal systolic diameter; LVAW,d, left ventricular end‐diastolic anterior wall thickness; LVPW,d, left ventricular end‐diastolic posterior wall; LVFS, left ventricular fractional shortening; LVEF, left ventricular ejection fraction.

Detecting the blood pressure levels in the tail artery of mice at Week 5 of modelling (Figure 2H–J), both systolic blood pressure (SBP) and diastolic blood pressure (DBP) in the tail artery of mice in the HFpEF group were significantly higher than those in the control group (SBP: Chow 92.50 ± 2.12 vs. HFpEF 125.25 ± 2.01; DBP: Chow 66.67 ± 2.44 vs. HFpEF 89.95 ± 1.67), all results were statistically different. The above results suggest that the HFpEF mixed diet leads to elevated blood pressure in mice.

Based on the above data, we confirmed that the HFpEF diet gradually induced diastolic function impairment in mice, and the diastolic function impairment phenotype was more significant and stable by Week 5, but did not lead to changes in cardiac systolic function in mice. In addition, the HFpEF diet also induced obesity and hypertension in mice, so this model can better simulate the clinical signs of HFpEF.

### 3.2 HBI‐8000 treatment improves diastolic dysfunction in mice with HFpEF

C57BL/6N male mice at 7–8 weeks of age were selected and randomly given Chow and HFpEF diets for 5 weeks of induction, and then each group was randomly divided into HBI‐8000 or 0.9% NaCl groups. Following 4 weeks of consecutive gavage, relevant functional tests and tissue samples were obtained. The overall experimental design consisted of four groups: Chow + 0.9% NaCl, Chow + HBI‐8000, HFpEF + 0.9% NaCl and HFpEF + HBI‐8000.

**FIGURE 3:**
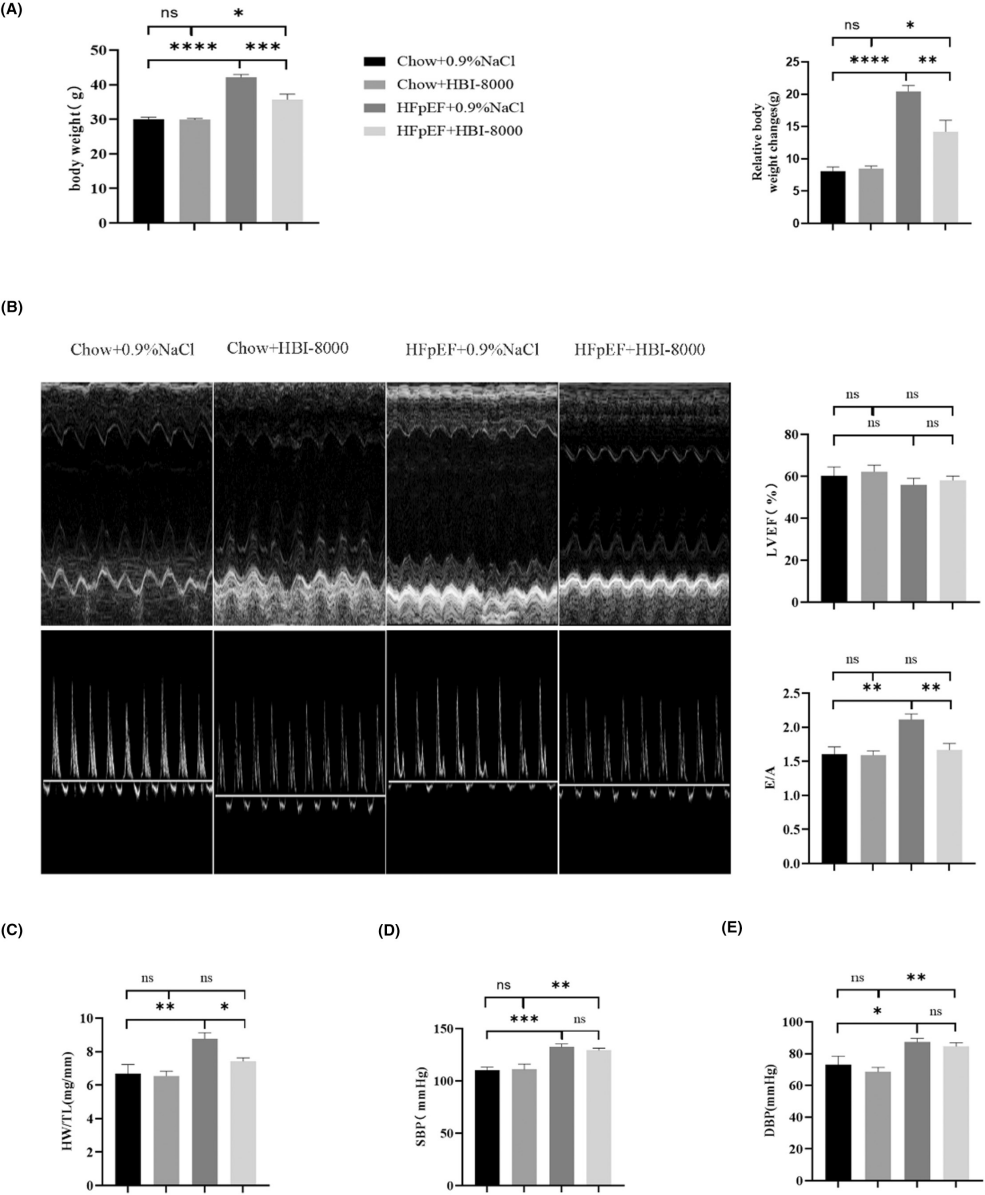
Results of functional index detection after 4 weeks of HBI‐8000 treatment. (A) Body weight levels of mice and relative body weight changes in each group after 4 weeks of drug intervention. (B) Characteristic M‐mode images and pulse‐wave images as well as LVEF and E/A statistics of mice in each group after 4 weeks of HBI‐8000 treatment. (C) Heart weight/tibia length (HW/TL) statistics of mice in each group after 4 weeks of drug intervention. (D, E) Blood pressure levels in the tail artery of mice in each group after 4 weeks of drug intervention. *p < 0.05, **p < 0.01, ***p < 0.001, ****p < 0.0001.

During the 4 weeks of feeding, mice in each group were given different diets and weighed (Figure 3A). The results showed that there was no significant difference in body weight between mice in the Chow + 0.9% NaCl and Chow + HBI‐8000 groups. However, intervention with HBI‐8000 attenuated body weight gain in mice compared to the HFpEF + 0.9% NaCl group (42.13 ± 0.84 g vs. 35.75 ± 1.51 g). In addition, we compared the changes in relative body weights of mice in each group, further confirming that HBI‐8000 treatment can improve obesity in mice.

After 4 weeks of drug administration, echocardiography was used to assess the cardiac function of mice (Figure 3B; Table 3). According to the statistical analysis, there were no significant differences in LVFS and LVEF among the four groups of mice treated with the relevant drugs, suggesting that there was no significant change in systolic function. Among them, compared with the Chow group, the HFpEF + 0.9% NaCl mice had an increase in left ventricular end diastolic diameter (LVID, d), left ventricular mass (LV mass), left ventricular end diastolic anterior wall thickness (LVAW, d) and left ventricular end diastolic posterior wall thickness (LVPW, d), indicating a certain degree of myocardial hypertrophy (Table 3). Additionally, HFpEF + HBI‐8000 significantly improved diastolic impairment compared with HFpEF + 0.9% NaCl group, as evidenced by a drop in E/A. As shown above, treatment with HBI‐8000 for 4 weeks has little effect on the mouse's systolic function, but significantly improves its diastolic dysfunction.

**TABLE 3:** Cardiac ultrasound data in mice.

	Chow + 0.9% NaCl (*n* = 5)	Chow + HBI‐8000 (*n* = 5)	HFpEF + 0.9% Nacl (*n* = 10)	HFpEF + HBI‐8000 (*n* = 10)
4 week diet				
HR (bpm)	485.83 ± 16.89	483.02 ± 8.04	473.82 ± 13.80	485.95 ± 17.22
LVID,d (mm)	3.81 ± 0.17	3.88 ± 0.13	3.97 ± 0.16	3.83 ± 0.11
LVID,s (mm)	2.61 ± 0.22	2.60 ± 0.17	2.92 ± 0.18	2.67 ± 0.12
LVAW,d (mm)	1.11 ± 0.02	1.06 ± 0.11	1.13 ± 0.09	1.09 ± 0.08
LVPW,d (mm)	0.95 ± 0.06	0.92 ± 0.06	1.12 ± 0.07	1.01 ± 0.05
LV mass (mg)	168.02 ± 8.85	164.16 ± 14.62	188.07 ± 12.99	160.26 ± 7.59
LVFS (%)	31.90 ± 2.71	33.21 ± 2.25	27.03 ± 2.04	27.24 ± 1.49
LVEF (%)	60.32 ± 3.40	62.24 ± 2.54	52.76 ± 3.15	55.02 ± 2.60
Peak mitral E velocity (mm/s)	706.90 ± 43.65	698.09 ± 51.39	760.67 ± 24.78	652.29 ± 25.83^#^
Peak mitral A velocity (mm/s)	448.84 ± 44.74	463.97 ± 39.60	354.99 ± 13.50	404.06 ± 22.64
Mitral E/A	1.61 ± 0.10	1.59 ± 0.06	2.10 ± 0.05**	1.65 ± 0.10^###^

*Note*: **p < 0.01 versus Chow + 0.9% NaCl group.

^#^ *p* < 0.05; ^###^ *p* < 0.001 versus HFpEF + 0.9% NaCl group.

The heart weight/tibia length (HW/TL) of mice after 4 weeks of HBI‐8000 administration was measured (Figure 3C). Based on the results, the HFpEF + 0.9% NaCl group (8.76 ± 0.44 mg/mm) was significantly higher than the Chow + 0.9% NaCl group (6.68 ± 0.54 mg/mm), which was partially improved by HBI‐8000 treatment (7.38 ± 0.27 mg/mm). As a result, we concluded that HBI‐8000 treatment improved cardiac hypertrophy in mice.

The blood pressure levels in the tail arteries of mice were examined after 4 weeks of treatment with HBI‐8000 (Figure 3D,E). According to the statistical data, HBI‐8000 slightly reduced blood pressure in the caudal artery of mice in the HFpEF group, but the results were not statistically significant. Therefore, the therapeutic effect of HBI‐8000 on HFpEF did not depend on the reduction in blood pressure, and its therapeutic effects were independent of blood pressure regulation.

Accordingly, the above data from animal experiments and functional tests in mice confirm that HBI‐8000 treatment reduces body weight and improves diastolic function of the heart in HFpEF mice.

### 3.3 Inhibition of cardiac hypertrophy and antifibrotic effects of HBI‐8000 in HFpEF mice

**FIGURE 4:**
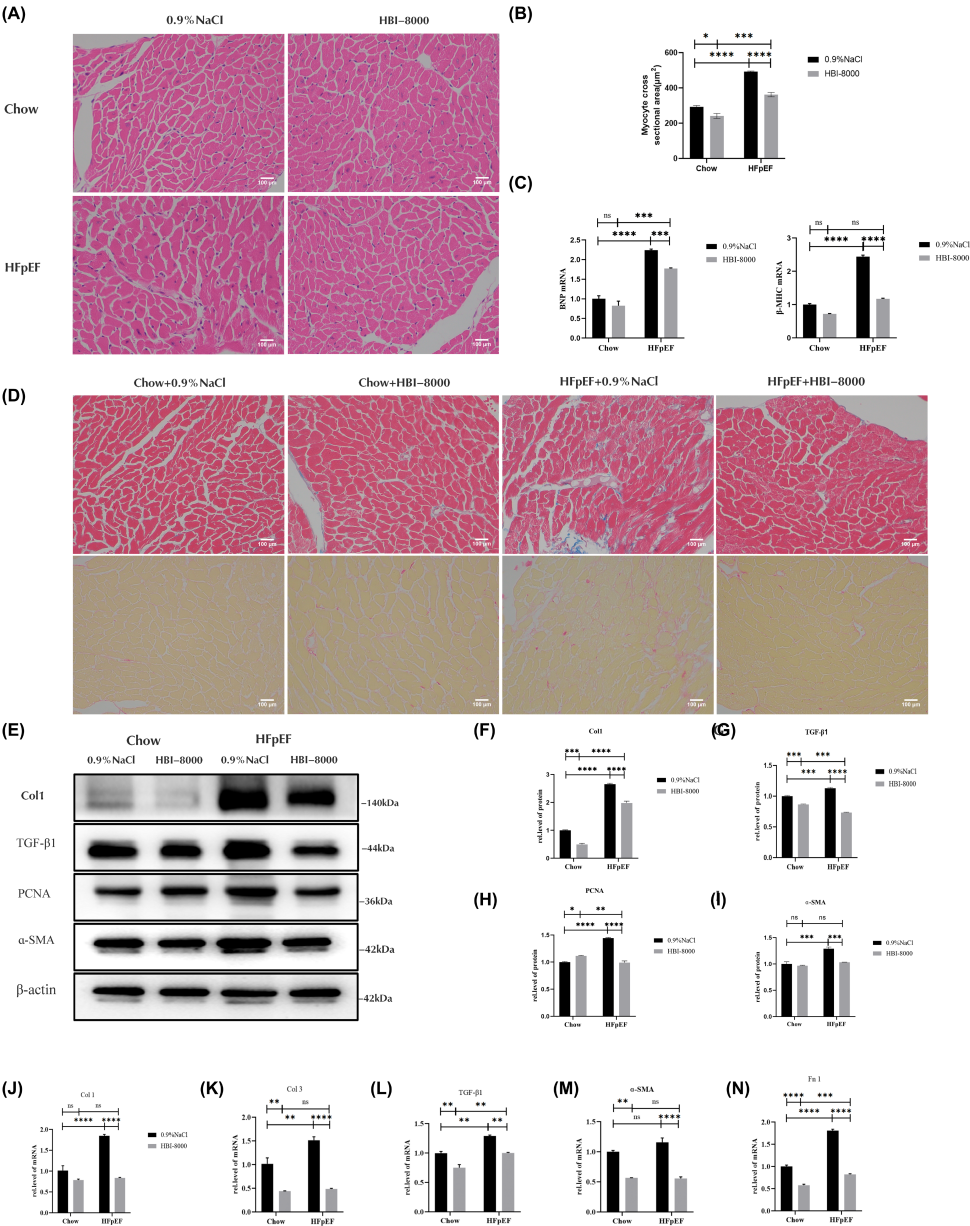
HBI‐8000 inhibits cardiac hypertrophy and ventricular fibrosis. (A, B) HE staining (Scale bar = 100 μm). (C) Relative expression of cardiac hypertrophy‐associated factor mRNA. (D) Masson staining in the upper half and PSR staining in the lower half (Scale bar = 100 μm). (E–I) Western blotting to detect the levels of myocardial fibrosis markers Col1, TGF‐β1, PCNA and ɑ‐SMA. (J–N) Levels of a‐SMA, Fn1, Col1, Col3 and TGF‐β1 were detected by qPCR. *p < 0.05, **p < 0.01, ***p < 0.001, ******p < 0.0001.

We examined the cardiac hypertrophy and fibrosis indexes in mouse left ventricular tissues in the present study. The area of cardiomyocytes was observed using HE staining (Figure 4A,B), and compared to the Chow group, the area of cardiomyocytes and the cross section of myocardial fibres were thicker in the HFpEF + 0.9% Nacl group. In contrast, HBI‐8000 treatment partially improved cardiomyocyte hypertrophy. As a further demonstration of whether drug treatment improved cardiac hypertrophy, LV tissue RNA was extracted to detect cardiomyocyte hypertrophy‐related indexes (Figure 4C). At the mRNA level, BNP and β‐MHC expression had different degrees of elevation in the HFpEF + 0.9% NaCl group, and BNP and β‐MHC transcript expression was down after administration of HBI‐8000 treatment.

We stained left ventricular tissues with Masson and PSR to determine the degree of left ventricular fibrosis (Figure 4D; Figure S4). According to the results, HFpEF + 0.9% NaCl induced significantly greater left ventricular fibrosis than Chow + 0.9% NaCl, and HBI‐8000 significantly reduced the degree of left ventricular fibrosis. To further confirm whether the drug had an inhibitory effect on myocardial fibrosis, we extracted left ventricular tissue proteins to detect the expression of myocardial fibrosis‐related proteins using western blot (Figure 4E–I), and compared with the Chow + 0.9% NaCl group, PCNA (a protein involved in the process of cell proliferation and DNA synthesis) and fibrosis markers in the HFpEF + 0.9% NaCl group (including Col1, TGF‐β1 and ɑ‐SMA) were elevated to different degrees. After the administration of HBI‐8000, fibrosis factor expressions were all decreased to different degrees. Furthermore, we extracted RNA from left ventricular tissue and observed the same results (Figure 4J–N).

The above experimental results indicate that the HFpEF group experienced different degrees of myocardial hypertrophy and fibrotic changes in comparison to the Chow group, and after 4 weeks of treatment with HBI‐8000, the overall degree of ventricular fibrosis and myocardial hypertrophy improved. Thus, we will utilize AngII to construct an in vitro fibrosis model in order to demonstrate the inhibitory effect of HBI‐8000 on myocardial fibrosis and to determine its potential mechanism of action.

### 3.4 HBI‐8000 treatment inhibits AngII‐induced cardiac hypertrophy and CFs proliferation

To clarify the effect of HBI‐8000 on cardiac hypertrophy, we pretreated with 5 μM HBI‐8000 for 1 h, then stimulated with AngII (100 and 500 nM) for 24 h, and determined the surface area of cardiomyocytes using immunofluorescence staining (Figure S1A,B). The results showed that AngII significantly increased the surface area of cardiomyocytes compared with the control group, whereas the surface area of cells decreased after administration of the drug treatment. In addition, we extracted RNA to detect the level of cellular hypertrophic factor expression in each group (Figure S1C). The results showed that 100 nM AngII and 500 nM AngII treatments increased the expression of ANP, β‐MHC and ɑ‐SKA in a concentration‐dependent manner, and the expression of the above factors decreased to different degrees after administration of HBI‐8000. Thus, we can demonstrate that HBI‐8000 treatment has a significant inhibitory effect on AngII‐induced cardiac hypertrophy.

**FIGURE 5:**
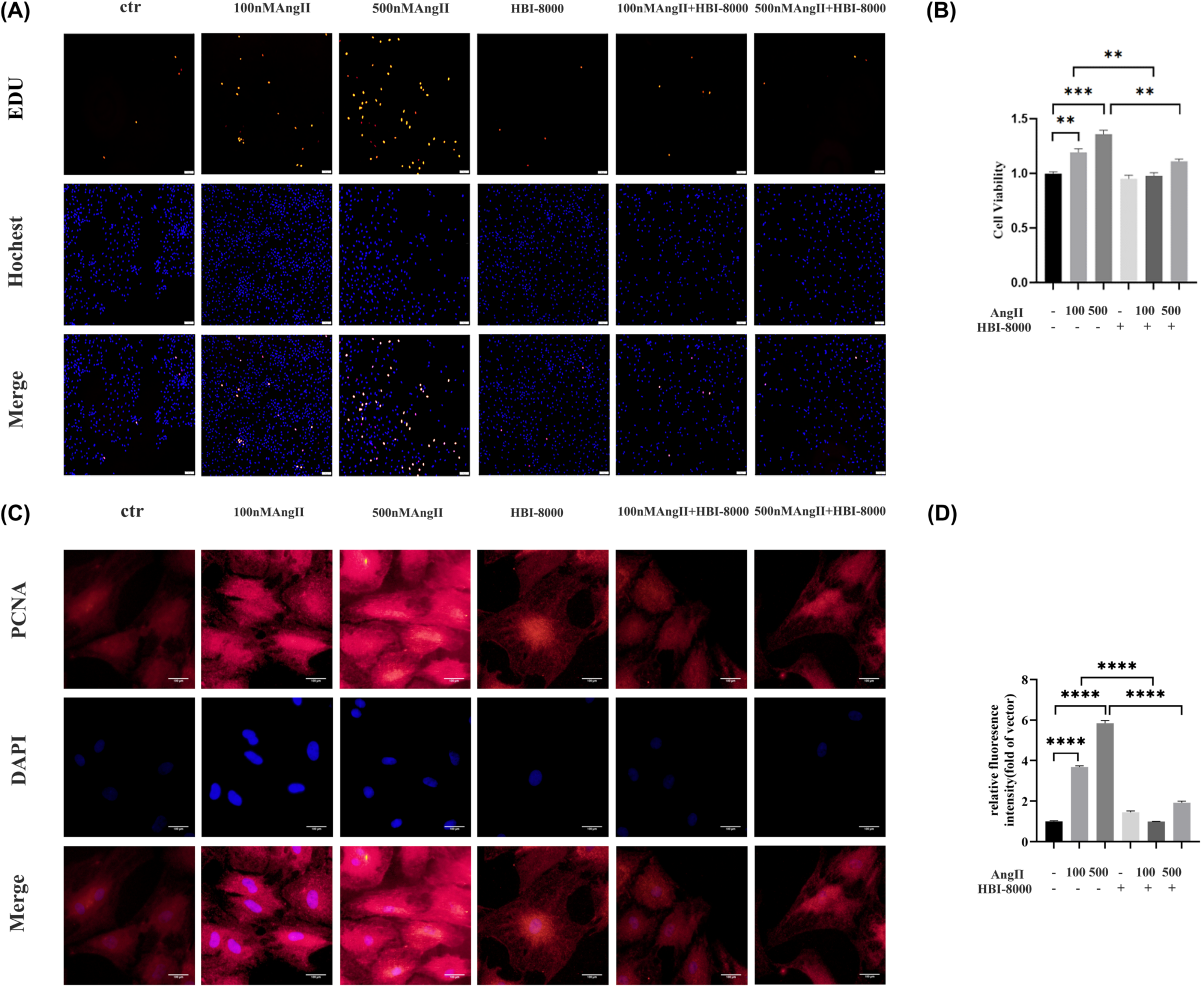
HBI‐8000 inhibits AngII‐induced CFs proliferation. (A) EDU kit to detect CFs proliferation (Scale bar = 50 μm). (B) CCK‐8 kit to detect CFs proliferation. (C, D) Immunofluorescence was used to determine the PCNA expression level of cells in each group (Scale bar = 100 μm). **p < 0.01, ***p < 0.001, ****p < 0.0001.

In order to investigate the effect of HBI‐8000 on myocardial fibrosis, we examined the proliferation of CFs using EDU and CCK‐8. In this study, we pretreated with 5 μM HBI‐8000 for 1 h and stimulated with 100 and 500 nM AngII for 24 h. Results indicated that AngII induced proliferation of CFs in a dose‐dependent manner, and that HBI‐8000 significantly reduced this proliferation (Figure 5A,B).

By using immunofluorescence to detect the expression of PCNA we were able to further confirm the inhibitory effect of HBI‐8000 on the proliferation of CFs. The results showed that AngII elevated PCNA expression, whereas HBI‐8000 treatment resulted in a significant decrease in PCNA expression levels (Figure 5C,D). In line with the results from EDU and CCK‐8, HBI‐8000 significantly inhibited the proliferation of CFs.

### 3.5 HBI‐8000 inhibits CFs differentiation

**FIGURE 6:**
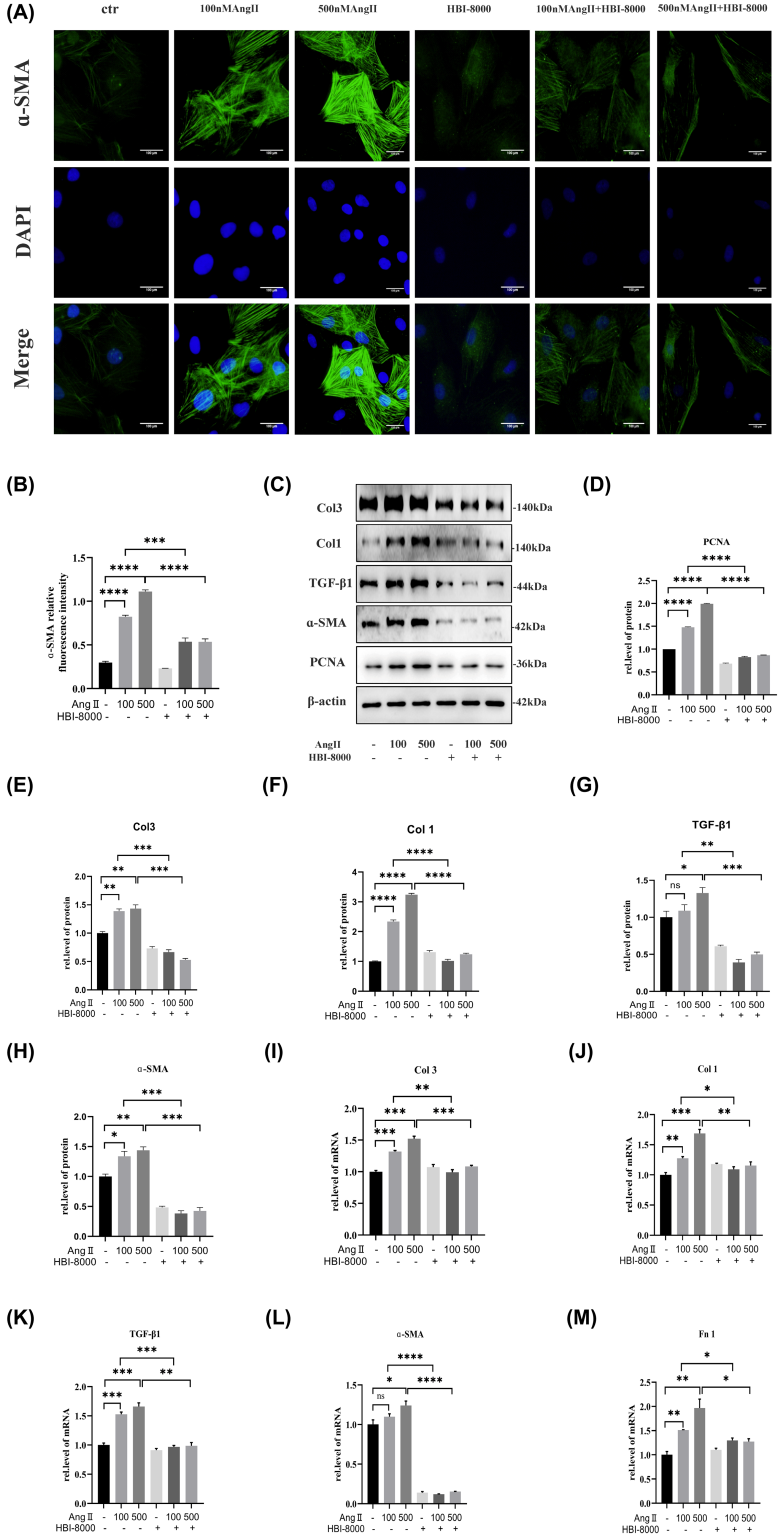
HBI‐8000 inhibits CFs differentiation. (A, B) Immunofluorescence was performed to determine the level of ɑ‐SMA expression in each group of cells (Scale bar = 100 μm). (C–H) Western blot was used to detect the expression levels of PCNA and cardiac fibrosis markers Col1, Col3, TGF‐β1 and ɑ‐SMA expression levels. (I–M) qRT‐RCR Western blot to detect the expression levels of cardiac fibrosis markers Col1, Col3, TGF‐β1, Fn1 and ɑ‐SMA. *p < 0.05, **p < 0.01, ***p < 0.001, *****p < 0.0001.

Immunofluorescence was used to detect ɑ‐SMA levels (Figure 6A,B). The results indicated that AngII increased ɑ‐SMA expression, while HBI‐8000 treatment significantly reduced AngII‐induced ɑ‐SMA expression. We performed Western blots to further investigate the inhibitory effect of HBI‐8000 on AngII‐induced differentiation of CFs. The results showed that HBI‐8000 inhibited AngII‐induced PCNA, Col1, Col3, TGF‐β1 and ɑ‐SMA expression (Figure 6C–H). Additionally, we performed qRT‐RCR, which confirmed that HBI‐8000 inhibited the expression of AngII‐induced fibrosis factor (Figure 6I–M).

### 3.6 HBI‐8000 exerts antifibrotic effects via the TGF‐β1/MAPK pathway

By Western blot and qRT‐RCR, we found that the expression level of TGF‐β1 was elevated in the HFpEF group, and HBI‐8000 treatment caused a significant decrease.

**FIGURE 7:**
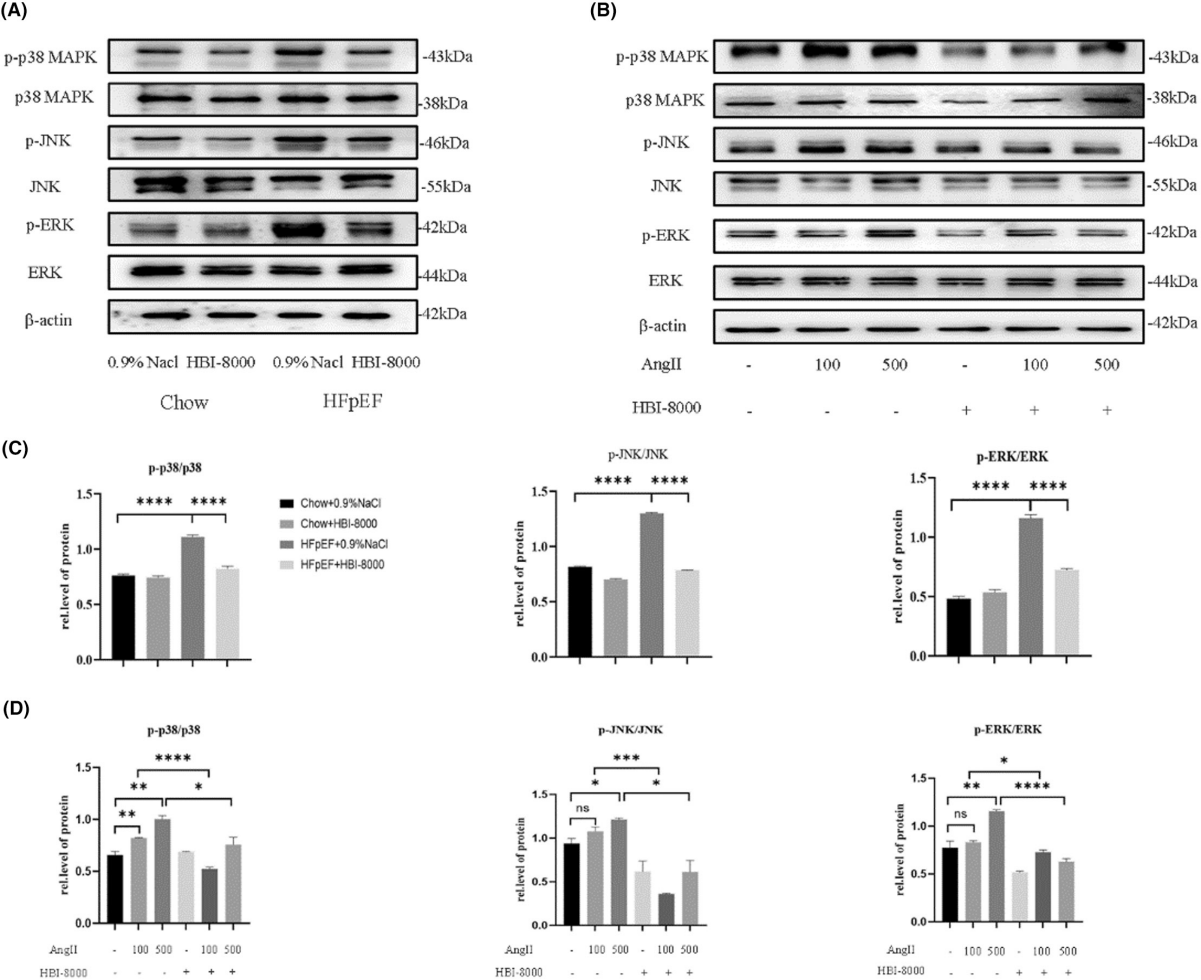
HBI‐8000 inhibited the activation of TGF‐β1/MAPK pathway. (A–D) Relative expression levels of p‐ERK, p‐JNK and p‐p38 MAPK proteins in animal tissues and cells were revealed by Western blotting and analysed using Image J. *p < 0.05, **p < 0.01, ***p < 0.001, ****p < 0.0001.

To clarify whether the antifibrotic effect of HBI‐8000 was related to the TGF‐β1/MAPK pathway, we detected the phosphorylation levels of ERK, JNK and p38 MAPK in left ventricular tissues using Western blot (Figure 7A,C). It was observed that the expression of p‐ERK, p‐JNK and p‐p38 MAPK was higher in the HFpEF + 0.9% NaCl group than in the Chow + 0.9% NaCl group. While, after treatment with HBI‐8000, the expression of all of these factors decreased in varying degrees. Similar results were also observed at the cellular level. The CFs were pretreated with 5 μM HBI‐8000 for 1 h, then stimulated with AngII at 100 and 500 nM for 24 h. Western blots were carried out to measure the phosphorylation levels of p38MAPK, ERK and JNK in each group of cells (Figure 7B,D). The results showed that AngII upregulated the expression of p‐p38MAPK, p‐ERK and p‐JNK, which was significantly reduced by HBI‐8000 treatment. Furthermore, SB203580 (p38MAPK inhibitor) pretreatment significantly reduced the expression of myocardial fibrosis markers (Figure S2). Thus, we further demonstrated that HBI‐8000 could alleviate cardiac fibrosis by inhibiting AngII‐induced activation of the TGF‐β1/MAPK pathway.

### 3.7 HBI‐8000 inhibits ROS accumulation

After Ang II stimulation, the production of ROS increased, but this effect was inhibited by HBI‐8000 (Figure S3A). By Western blotting, we found that AngII increased the expression of p‐smad2/3 in CFs, and the expression of p‐smad3 was decreased after HBI‐8000 treatment, while p‐smad2 was not significantly changed (Figure S3B).

## 4 DISCUSSION

Heart failure (HF) is a major public health problem affecting millions of people worldwide.^20^ The latest guidelines classify HF according to LVEF as follows: heart failure with reduced ejection fraction (HFrEF): LVEF ≤40%; heart failure with improved ejection fraction (HFimpEF): first classified as HFrEF with a re‐measured LVEF >40%; heart failure with mildly reduced ejection fraction (HFmrEF): LVEF of 41%–49% with evidence of increased LV filling pressure and HFpEF: LVEF ≥50% with evidence of increased LV filling pressure.^21^ Of these, HFpEF accounts for approximately half of the total heart failure cases and its occurrence is associated with an increase in major risk factors such as increasing age, hypertension, diabetes, obesity and atrial fibrillation.^22^, ^23^ Current domestic and international guidelines in the treatment of HFpEF mainly recommend traditional RAAS system inhibitors, diuretics, β‐blockers or antihypertensive, glycaemic control and exercise for its complications, supplemented by adjuvant treatments such as anti‐atrial fibrillation, prevention of thrombosis and maintenance of renal function.^21^, ^24^, ^25^, ^26^, ^27^ However, common clinical therapeutic agents such as ACEI/ARBs and diuretics, although effective in HFrEF, have limited benefit for patients with HFpEF.^28^, ^29^ SGLT2i (dapagliflozin, empagliflozin, etc.), which inhibits the opening of sodium‐glucose transporter protein 2 channels, is by far the most promising therapeutic agent in the field of HFpEF research. Studies have shown that dapagliflozin and empagliflozin lead to a reduced risk of the composite endpoint event of hospitalisation for heart failure or cardiovascular death compared with placebo, which is driven by hospitalisation for heart failure but not cardiovascular mortality.^30^, ^31^ Therefore, it is particularly important to explore novel therapeutic agents for patients with HFpEF. As HFpEF progresses, the severity of cardiac hypertrophy and fibrosis increases, and its severity is strongly correlated with disease prognosis.^32^ Therefore, improving cardiac fibrosis is important to inhibit the progression of HFpEF.

It is thought that HDAC is capable of re‐dense chromatin structure and inhibit the synthesis of mRNA by removing acetylated residues from lysine residues. Depending on their cellular localisation, HDAC are divided into four major groups: class I, class II, class III (Sirtuin family) and class IV (Table S1). In Classes I, II and IV, zinc molecules serve as activators, while in Class III, NAD+/NADH molecules act as activators because of the conservation of the catalytic core structural domain.^33^ Of these, Class II can be further divided into two subgroups: Class IIa, which has a C‐terminus, and Class IIb, which has two deacetylase structural domains. Currently, six HDACi have been approved for marketing by the US Food and Drug Administration (FDA): vorinostat (SAHA), beli‐nostat (PXD101), pabilostat (LBH589), romidepsin (FK228), HBI‐8000 and mocetinostat (MGCD0103), which are primarily used as treatments for cancers including T‐cell lymphomas, B‐cell lymphomas, multiple myelomas and prostate cancer.^34^ Among them, HBI‐8000 is a subtype‐selective benzamide HDACi, mainly targeting subtypes 1, 2 and 3 in Class I HDACs and subtype 10 in Class IIb, and is a broad‐spectrum anti‐tumour drug independently developed in China. Studies have shown that the drug mainly acts through inducing cell cycle block, promoting tumour cell differentiation, inducing tumour cell apoptosis and autophagy.^35^, ^36^

Among all classes of HDACs, Class I and Class II HDACs are involved in the regulation of cardiac hypertrophy, but they play opposite roles. Numerous studies have shown that Class I HDACs 2, 3, and 8 have a role in promoting cardiac hypertrophy.^37^, ^38^, ^39^ In contrast, class IIa HDAC inhibits cardiac hypertrophy by suppressing cardiac‐specific transcription factors, such as cellular enhancer factor 2 (MEF2), GATA‐binding protein 4 and activated T‐cell nuclear factor.^40^, ^41^ In addition, the regulation of cardiopulmonary fibrosis by HDAC has been previously reported in the literature, therefore, the antifibrotic ability of HDACi has been widely appreciated.^42^, ^43^ It has been shown that SAHA inhibits MFs production by improving myoendoplasmic reticulum Ca^2+^‐ATPase activity in cardiomyocytes mainly through inhibition of class I HDAC.^44^ Similarly, MGCD0103 selectively inhibits HDAC1 and HDAC2, inhibits IL‐6/STAT3 signalling thereby reducing inflammatory cell infiltration and ameliorating interstitial fibrosis.^45^ Taken together, the above studies now suggest that class I HDACs are pro‐hypertrophic and fibrotic factors in cardiac tissues, and selective inhibition or pan‐inhibition of the above HDACs may serve to ameliorate cardiac hypertrophy and fibrosis. In the present study, we demonstrated that HBI‐8000 ameliorated cardiac hypertrophy and fibrosis in HFpEF mice. Therefore, in the following, we aimed to investigate the specific mechanism of action of HBI‐8000.

AngII is a major inducer of RAAS, and as a traditional activator of cardiac fibrosis, it promotes the conversion of CFs to MFs which leads to the deposition of ECM.^46^ In vivo, TGF‐β1 is an important factor that has been clearly identified as inducing fibrotic responses, and the MAPK pathway is atypical of its downstream pathway. It has been demonstrated that TGF‐β1 stimulates the proliferation and differentiation of CFs,^47^ which are activated in cardiac diseases to encourage cardiac repair and fibrotic remodelling.^48^ There is evidence that p38MAPKs positively regulate collagen and ECM production in dermal fibroblasts.^12^, ^49^ Additionally, JNK and ERK signalling pathways have been linked to fibrosis.^50^, ^51^, ^52^ In this study, we investigated whether HBI‐8000 exerts its antifibrotic effects through the TGF‐β1/MAPK signalling pathway.

ROS is one of the important intermediate oxides during oxidative stress and plays an important role in various cardiovascular diseases.^53^ Ang II stimulation can lead to ROS accumulation and thus activate the ROS/TGFβ/Smad2/3 signalling pathway leading to cardiac fibrosis.^54^ In the present study, we observed that Ang II stimulation led to an increase in ROS accumulation and p‐smad2/3 expression, whereas HBI‐8000 treatment reduced ROS generation and p‐Smad3 expression. It was shown that smad7 inhibits TGF‐β1‐mediated smad2/3 phosphorylation and negatively regulates TGF‐β1‐induced fibrosis.^55^ And HDAC3 is involved in maintaining the basal inhibitory state of smad7.^56^ In addition, smad3 has been reported to inhibit HDAC3‐mediated progressive fibrosis by regulating the expression of PDCD5 (programmed cell death 5) in CFs.^57^

By inducing metabolic disorders and hypertension, the ‘Two‐Hit’ HFpEF mouse model is stable and easy to construct.^19^ In the present study, we demonstrated that the HFpEF diet gradually induced diastolic dysfunction in mice, and this phenotype was more significant and stable by Week 5, without altering cardiac systolic function. In addition, the HFpEF diet also induced obesity and hypertension in mice, so this modelling method can better model the clinical signs of HFpEF. When mice were treated with HBI‐8000 for 4 weeks, the body weight, HW/TL and E/A decreased significantly compared to the model group, but the blood pressure did not change significantly. The above results suggest that HBI‐8000 improves diastolic dysfunction, and its therapeutic effect is independent on blood pressure regulation.

In this study, we applied HE staining, Masson staining, and PSR staining to confirm that cardiac hypertrophic and fibrotic alterations did exist in the hearts of HFpEF mice. In accordance with the pathological results, we obtained left ventricular tissue proteins and mRNA from mice, and found that cellular hypertrophic factors and fibrosis‐related factors were elevated in the HFpEF group, whereas HBI‐8000 administration reduced their expression. Based on these findings, HBI‐8000 is effective in ameliorating the symptoms of HFpEF such as myocardial fibrosis and cardiac hypertrophy.

At the cellular level, we stimulated NRCMs and CFs with AngII and showed that HBI‐8000 inhibited AngII‐induced cardiomyocyte hypertrophy and CFs proliferation and differentiation, and down‐regulated the expression levels of ANP, β‐MHC and ɑ‐SKA as well as fibrosis‐related factors. In addition, we found that the HFpEF group and AngII stimulated groups significantly increased the expression of TGF‐β1 as well as phosphorylated p38MAPK, JNK, and ERK, whereas the drug‐treated group dramatically decreased the expression of the above factors. It suggests that HBI‐8000 may exerts its antifibrotic effect by inhibiting the activation of the TGF‐β1/MAPK pathway. Consistent with these results, we observed that SB203580 (p38MAPK inhibitor) treatment significantly inhibited AngII‐induced elevated levels of cardiac fibrosis markers. All in all, our results suggest that HBI‐8000 can block AngII‐induced conversion of CFs to MFs, which may be achieved by inhibiting the activation of the TGF‐β1/MAPK pathway. However, our study has several limitations. As the experimental conditions were limited, we were unable to perform further examinations of cardiac diastolic function and exercise tolerance to confirm the multiple ameliorative effects of the studied drugs on the animal model.

## 5 CONCLUSION

In conclusion, our results suggest that HBI‐8000 improves HFpEF by blocking the TGF‐β1/MAPK signalling pathway and inhibiting the proliferation and transformation of CFs as well as the excessive deposition of ECM.

## AUTHOR CONTRIBUTIONS

**Guohai Su:** Conceptualization (lead); project administration (lead); supervision (supporting); visualization (equal); writing – review and editing (supporting). **Jing Tian:** Methodology (lead); resources (lead); writing – original draft (lead). **Wenjing Li:** Data curation (equal); formal analysis (equal); resources (equal); software (equal); validation (equal); writing – review and editing (equal). **Lu Zeng:** Data curation (supporting); software (supporting). **Yang Li:** Data curation (supporting); methodology (supporting); software (supporting). **Jiamin Du:** Data curation (supporting); resources (supporting); software (supporting). **Ying Li:** Conceptualization (supporting); formal analysis (supporting); project administration (supporting); supervision (supporting); writing – review and editing (supporting). **Bin Li:** Conceptualization (equal); formal analysis (supporting); funding acquisition (supporting); project administration (equal); writing – review and editing (equal).

## CONFLICT OF INTEREST STATEMENT

The authors declared no conflicts of interest.

## Supporting information


Figures S1–S4

Table S1

## Data Availability

The data from public database supporting the findings of this study are available in the methods of this article. All raw data of this study are available on request from the corresponding author.
