# Supplementary material for: HBI‐8000 improves heart failure with preserved ejection fraction via the TGF‐β1/MAPK signalling pathway
Source: J Cell Mol Med. 2024 Mar 20;28(7):e18238. doi: 10.1111/jcmm.18238 (PMC10955178; doi:10.1111/jcmm.18238)
Supplement: Supplementary file 1 — Figures S1–S4 [file JCMM-28-e18238-s001.docx]

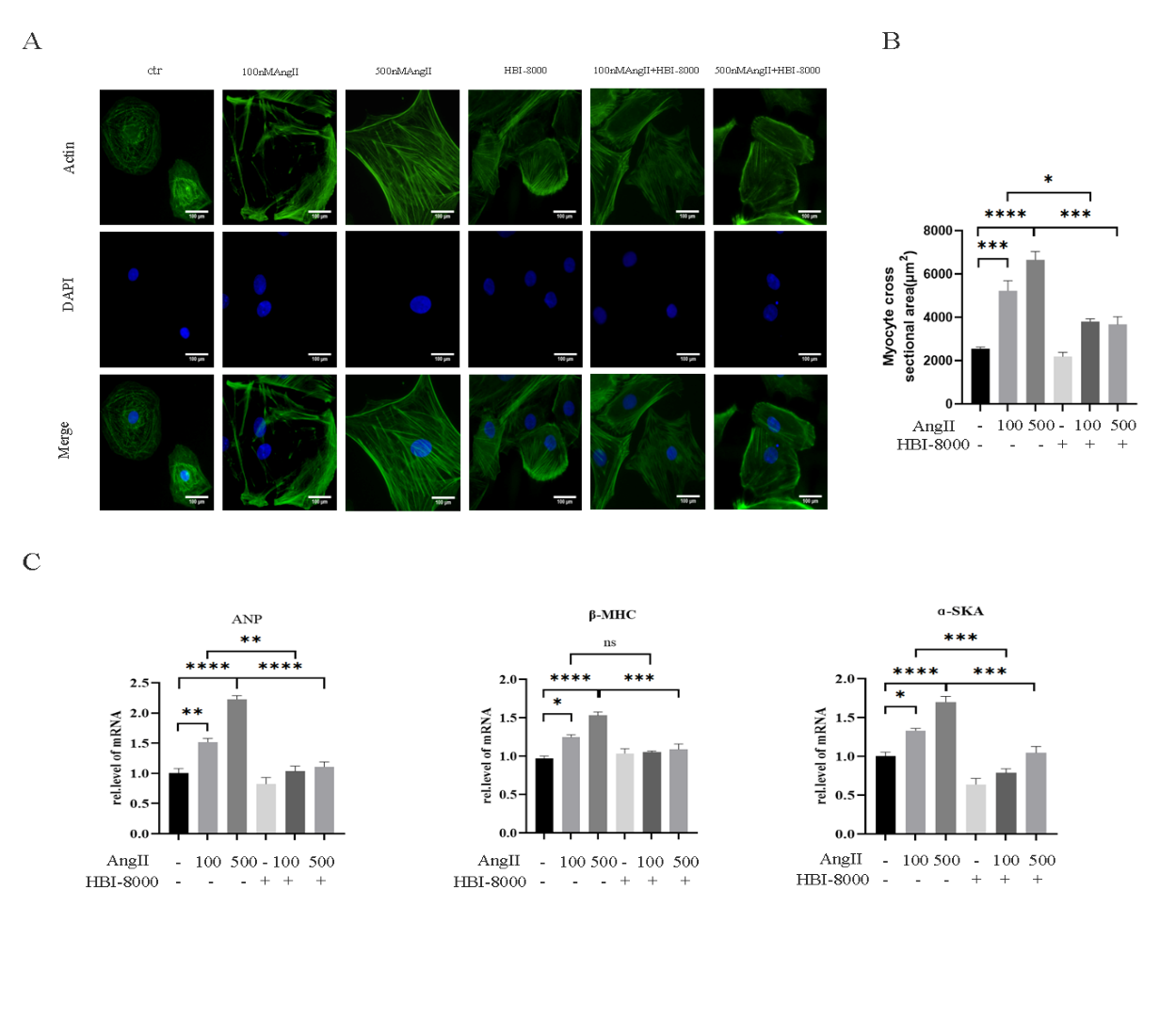


**Supplementary Figure 1.** **HBI-8000 inhibited AngII-induced cardiomyocyte hypertrophy.**





**Supplementary Figure 2.** **Detection of cardiac fibrosis indices following SB203580 treatment using Western blot and qRT-PCR.**


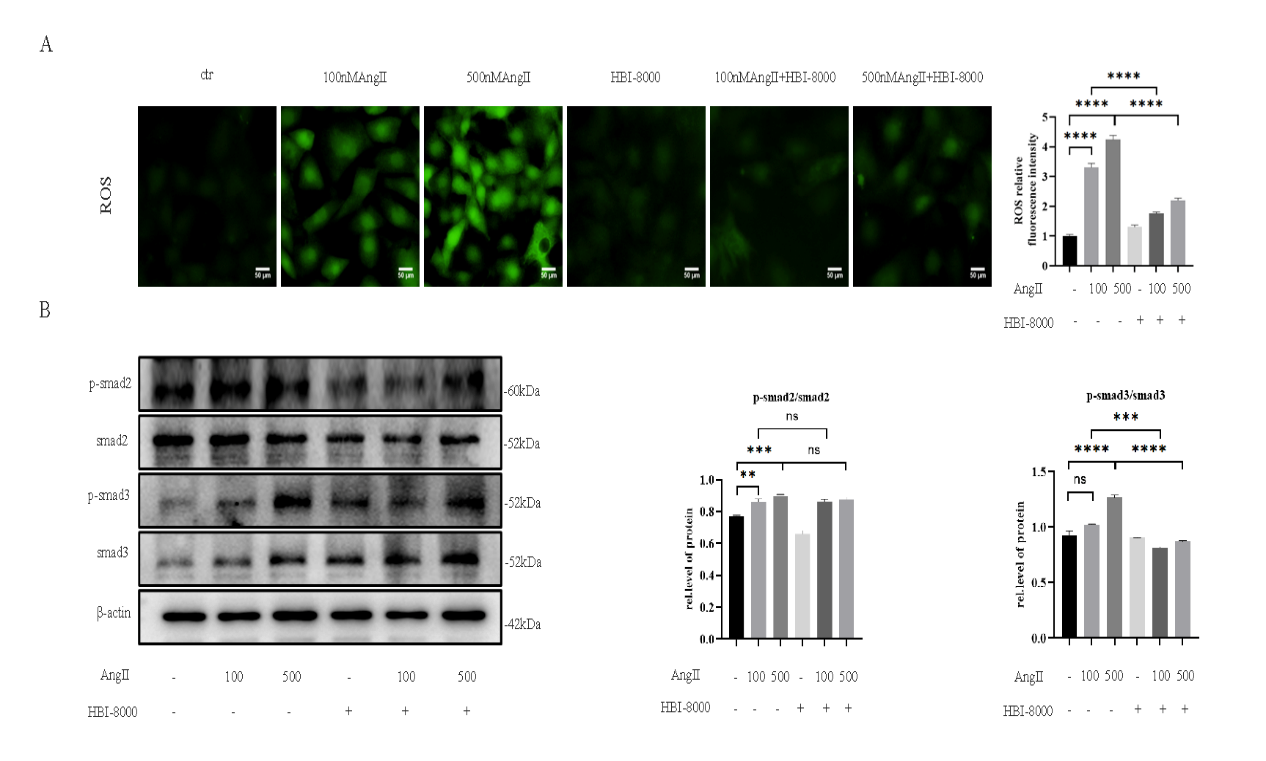


**Supplementary Figure 3.** **HBI-8000 inhibits ROS accumulation and smad signalling pathway activation.**


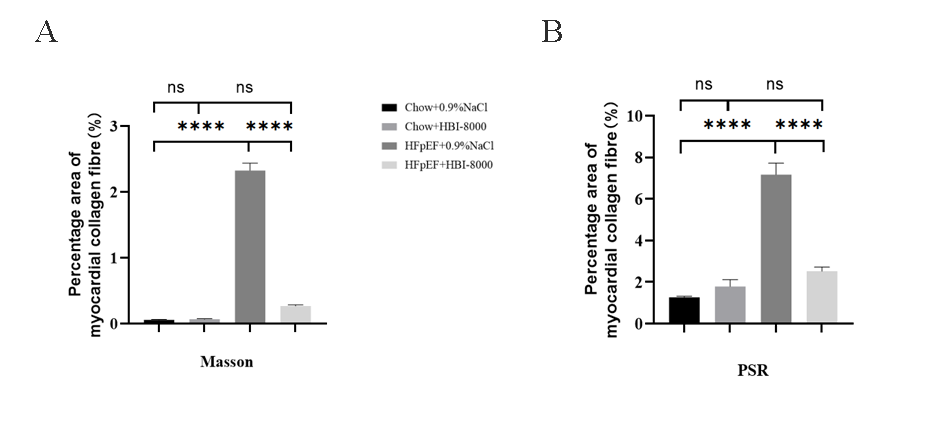


**Supplementary Figure 4. HBI-8000 inhibits myocardial fibrosis.**
