# Supplementary material for: HBI‐8000 improves heart failure with preserved ejection fraction via the TGF‐β1/MAPK signalling pathway
Source: J Cell Mol Med. 2024 Mar 20;28(7):e18238. doi: 10.1111/jcmm.18238 (PMC10955178; doi:10.1111/jcmm.18238)
Supplement: Supplementary file 2 — Table S1 [file JCMM-28-e18238-s002.docx]

**Supplementary Table 1** Classification of HDACs

| HDACs | Classifications | Function |
| --- | --- | --- |
| Class Ⅰ HDACs | HDAC1、HDAC2、HDAC3、HDAC8 | Influences the proliferative activity of cells |
| Class ⅠIa HDACs | HDAC4、HDAC5、HDAC7、HDAC9 | Involved in cell differentiation |
| Class ⅠIb HDACs | HDAC6、HDAC10 |  |
| Class III HDACs  (or Sirtuins) | SIRT1、SIRT2、SIRT3、SIRT4、SIRT5、SIRT6、 SIRT7 | Regulates monocyte apoptosis |
| ClassIV HDACs | HDAC11 | - |
